# Conducting Prolonged General Anesthesia without Intravenous Access in a Child with Hypoplastic Left Heart Syndrome

**DOI:** 10.1155/2017/5604975

**Published:** 2017-10-17

**Authors:** Phat T. Dang, Binjon Sriratana

**Affiliations:** Department of Anesthesiology, Rush University Medical Center, 1653 W. Congress Parkway, Jelke 7, Chicago, IL 60612, USA

## Abstract

Children with chronic medical conditions often need multiple intravenous (IV) access instances during their hospitalizations, both peripheral and central. Obtaining a working IV in this patient population undergoing general anesthesia can be challenging. In our case report, we describe a method of administering general anesthesia in an infant with partially repaired hypoplastic left heart syndrome without IV access.

## 1. Introduction

Establishing intravenous (IV) access is customary for patients undergoing general anesthesia, especially in the pediatric population [1]. However, obtaining IV access in critically ill infants and children can be very difficult. These patients usually require multiple IV punctures during their hospital course, which leads to deterioration of access sites and often necessitates central venous cannulation [2, 3]. General anesthesia without IV access is safe for healthy pediatric patients undergoing simple and brief procedures such as myringotomy tubes and ophthalmology exams [4–6]. However, it is not encouraged to anesthetize children with complex medical conditions without IV access, especially for long durations of general anesthesia. In this case report, we present an unconventional method of administering general anesthesia without IV access in a chronically ill infant with partially repaired hypoplastic left heart syndrome.

## 2. Case Description

The patient is a 12-month-old male with a history of hypoplastic left heart syndrome status after Norwood procedure and Sano and Glenn shunts, who presented to the pediatric intensive care unit in septic shock. His past medical history includes hypoxic ischemic encephalopathy, seizures, and developmental delay. His admission was further complicated by an ileal perforation, which required a small bowel resection and ileostomy. Given the complexity of his clinical presentation, need for volume assessment, and difficult IV access, he underwent multiple central venous catheter (CVC) and peripherally inserted central catheter (PICC) placements by the pediatric intensivists and surgeons at the following locations: right subclavian, right basilic, right saphenous, and left femoral veins. Either all attempts were unsuccessful or the catheters eventually failed. Later in his admission, the patient required an ileostomy reversal and BROVIAC catheter placement for total parental nutrition. One day prior to this scheduled operation, a left arm PICC was placed; however, it stopped working shortly afterwards. To make matters worse, his peripheral IV (PIV) infiltrated later that evening and subsequent attempts for a PIV in all the patient's extremities were unsuccessful. Since it was impossible to obtain IV access preoperatively, the patient was taken to the operating suite and induced via inhaled sevoflurane and oxygen. The pediatric cardiac anesthesiologist made three unsuccessful PIV attempts after induction. The patient was then given intramuscular (IM) atropine and intubated with an oral endotracheal tube via direct laryngoscopy without difficulty. The patient remained hemodynamically stable after induction and intubation. The case was turned over to the pediatric surgeons to attempt BROVIAC placement. Sevoflurane was titrated between 1.0 and 1.3%, and spontaneous ventilation was maintained with 70% inspired oxygen. His oxygen saturations (SpO_2_) were maintained between 80 and 88%. Fluctuations in blood pressure and heart rate were monitored for discomfort, and multiple doses of IM ketamine (0.5–1 mg/kg) were given periodically for sedation and analgesia.

Surgical exposure and cannulation of the right saphenous, both femoral veins, left axillary, and left external jugular veins were unsuccessful due to venous stenosis. Eventually, after 4.5 hours of attempts, a very small left internal jugular (IJ) vein was identified and a BROVIAC catheter was successfully placed. The catheter was then given to the anesthesiologist and IV fluids and antibiotics were initiated. The ileostomy reversal was performed afterwards, and the patient was extubated at the end of the case. His vital signs were within normal limits throughout the procedure and he experienced no anesthetic or surgical complications.

## 3. Discussion

Obtaining IV access to conduct general anesthesia can be a challenge in many cases, especially in the pediatric population. Many anesthesiologists forego PIV access in healthy patients for outpatient procedures. It is common to conduct general anesthesia without PIV access in healthy children undergoing dental extractions, minor otolaryngology procedures, or ophthalmology exams under anesthesia [4–6]. However, the lack of IV access would make administering rapid acting medications during emergency situations more difficult. In patients with congenital heart diseases such as our patient, frequent hospitalizations, blood draws, and PIV attempts cause deterioration of their venous sites making PIV access a challenge. Low volume status is also a major contributor [7]. These patients usually need alternative methods to gain IV access. For example, Gleich and Nemergut advocate using a six-inch angiocatheter using Seldinger's technique in the forearm veins under ultrasound (US) guidance [7], while Johnson et al. reported the use of intraosseous (IO) access as a temporary bridge [8].

We realized establishing PIV access preoperatively would be futile given the multiple failed attempts by the ICU staff. US guidance was not utilized for our patient because the bilateral hematomas in the regions of the forearm, antecubital, and saphenous veins would make US visualization difficult. Therefore, we reviewed the child's medical and anesthetic records, conducted a careful airway exam, and assessed him to be euvolemic. The patient was safely inhalationally induced hoping to make PIV insertion easier afterwards. Unfortunately, it was still impossible to obtain PIV access despite multiple attempts by a pediatric cardiac anesthesiologist. After discussing with the facility's most experienced pediatric surgeon, the surgeon was confident they would be able to expose and cannulate a vessel. We acknowledged at this junction that IV access was highly unlikely by other methods, and a long term IV catheter was necessary for the care of this child. Therefore, the patient was intubated to allow the surgeon to place the BROVIAC catheter and utilize it for the remainder of the surgery. IO access was considered after the failed PIV attempts after induction. Given the risks of infection, infiltration, and interference with the operative field, it was decided to reserve IO placement in case of an intraoperative emergency. An IO kit was kept accessible for the surgeon or anesthesiologist for rapid insertion. Emergency drugs (i.e., epinephrine, atropine, and succinylcholine) were prepared to administer IM or through the endotracheal tube.

As single ventricle heart pathologies are repaired with systemic-to-pulmonary artery shunts, such as our patient's Glenn shunt, it is favorable to avoid cannulation of the superior vena caval system to prevent thrombosis and obstruction of pulmonary blood flow [1, 3]. However, avoiding the SVC vessels was not feasible. The surgeon could not thread catheters through the femoral, saphenous, axillary, and external jugular veins under direct visualization due to venous stenosis. Therefore, our patient was initially without any IV access for an unanticipated duration of 4.5 hours until the left IJ vein was finally cannulated as a last resort.

Lower concentrations of sevoflurane were administered to minimize its cardiac depressant effects [1]. We also maintained spontaneous ventilation to avoid an increase in intrathoracic pressure, maintain cardiac output, and preserve passive pulmonary arterial (PA) blood flow in respect to his Glenn shunt. However, hypercarbia and acidosis are also possible with prolonged spontaneous ventilation and detrimental in maintaining an adequate transpulmonary gradient [9]. Therefore, we monitored his end-tidal carbon dioxide closely, which never exceeded 50 mmHg. Repeated doses of IM ketamine were administered to supplement sevoflurane in order to provide an adequate level of general anesthesia and analgesia while preserving spontaneous ventilation. Its sympathomimetic effects were also beneficial in maintaining cardiac output and PA flow [1]. It must be noted that his preoperative echocardiogram showed a patent Glenn shunt and adequate ventricular function. Our patient was cardiopulmonary stable the entire duration of the case (i.e., no bradycardia, hypotension, hypoxia, etc.)

## 4. Conclusion

Obtaining IV access for general anesthesia in pediatric patients with congenital heart disease is strongly advised. However, when IV access is unobtainable, we highlight in our case that it is feasible to safely perform general anesthesia over an extended period of time in these patients. While we do not advise with using this method as routine practice, we encourage fellow anesthesiologists to be adaptable when faced with particular circumstances such as this. Extreme caution must be exercised when managing infants who have undergone complex cardiac repairs for surgeries without IV access.
